# The synergistic antitumor effect of IL-6 neutralization with NVP-BEZ235 in hepatocellular carcinoma

**DOI:** 10.1038/s41419-022-04583-5

**Published:** 2022-02-14

**Authors:** Yao Wang, Xiaolong Miao, Yuancong Jiang, Zelai Wu, Xuhang Zhu, Han Liu, Xiaoying Wu, Jinzhen Cai, Xianfeng Ding, Weihua Gong

**Affiliations:** 1 College of Life Sciences and Medicine, Zhejiang Sci-Tech University, Hangzhou, 310018 Zhejiang China; 2 Department of Surgery, Second Affiliated Hospital of School of Medicine, Zhejiang University, Hangzhou, 310058 China; 3 Department of head and neck Surgery, Zhejiang Cancer Hospital, Hangzhou, China; 4 Department of Thyroid and Breast Surgery, Tongde Hospital of Zhejiang Province, Hangzhou City, China; 5 Organ Transplantation Center, The Affiliated Hospital of Qingdao University, Qingdao, China; 6 Liver Disease Center, The Affiliated Hospital of Qingdao University, Qingdao, China; 7 Liangzhu Laboratory, Zhejiang University Medical Center, Hangzhou, 311121 China

**Keywords:** Targeted therapies, Liver cancer

## Abstract

Hepatocellular carcinoma (HCC) still ranks among the top cancers worldwide with high incidence and mortality. Due to abnormal activation of the PI3K/AKT/mTOR signalling pathway in HCC, targeting this pathway represents a potential therapeutic strategy. NVP-BEZ235 is a novel dual-targeted ATP-competitive PI3K/mTOR inhibitor that has shown effective antitumor effects. In this study, we found that interleukin-6 (IL-6) was significantly increased after exposure to NVP-BEZ235, and we proposed a treatment in which an anti-IL-6 antibody was combined with NVP-BEZ235 for HCC. In vitro results revealed that targeted inhibition of IL-6 potentiated the antitumor effects of NVP-BEZ235 in HCC cells. The mechanism might be attributed to their synergistic inhibitory activity on the PI3K/AKT/mTOR signalling pathway. Furthermore, an in vivo study demonstrated that combined administration of NVP-BEZ235 and anti-IL-6 Ab reduced HCC tumour load more effectively than either NVP-BEZ235 or anti-IL-6 Ab treatment alone. These findings add guidance value to the analysis of HCC and provide a reference for clinical treatment.

## Introduction

Hepatocellular carcinoma (HCC) accounts for one of the major causes of cancer-associated mortality worldwide, and the largest number of cases are in Asia [1]. In particular, HCC represents approximately 90% of all primary liver cancer cases globally [2]. Since HCC is difficult to diagnose during early stages and has a poor prognosis, the five-year survival of patients with HCC is not optimistic [3]. At present, radiofrequency ablation, hepatectomy, liver transplantation, and other local therapies are typically used for patients with early- and middle-stage liver cancer. Systemic therapy tends to be used for patients with advanced disease [4]. Sorafenib and lenvastinib are the primary first-line therapies at present [5, 6]. However, most drugs have not shown significant survival benefits in recent years [7]. At present, a new direction of HCC treatment includes the strategy of combination therapy using molecular targeted inhibitors, which can achieve improved synergy by enhancing the sensitivity of targeted drugs [7].

Previous studies have identified common mutations in the PI3K/AKT/mTOR signalling pathway, which is a typical pathway in the pathogenesis of HCC [8]. During the pathogenesis of HCC, overexpression of hepatocyte-specific PI3K can lead to steatosis and lipid accumulation, accelerating tumour formation [9, 10]. More specifically, PI3K/AKT/mTOR signalling levels induce the expansion of tumour-initiating cells by regulating cell cycle progression and are associated with recurrence and chemoresistance of hepatocellular carcinoma [11]. Targeted therapy for the PI3K/AKT/mTOR signalling pathway provides important treatment opportunities for patients with liver cancer [12]. At present, many molecular targeted therapeutic drugs have emerged that target different carcinogenic sites of the PI3K/AKT/mTOR signalling pathway [13–15]. NVP-BEZ235, a novel dual inhibitor of PI3K and mTOR, blocks the PI3K/AKT/mTOR signalling pathway. At present, a number of preclinical experiments have demonstrated that NVP-BEZ235 inhibits the proliferation of tumour growth [16, 17], and chemotherapeutic agents display significant synergistic effects with this drug [18–20].

In addition, the PI3K/AKT/mTOR signalling pathway plays a complex role in regulating the inflammatory response and maintaining the function of the host immune system [21]. Since the PI3K/AKT/mTOR signalling pathway is widely involved in the physiological and biochemical processes of cells, it is not surprising that molecular targeted drugs exhibit various toxicities. A related study found that NVP-BEZ235 enhanced the levels of IL-6 and TNF-α 24 h after administration in murine acute lung injury models [22]. In this study, we further examined the safety and efficacy of NVP-BEZ235 to improve its efficacy.

## Materials and methods

### Cell culture and transfection

The HCC cell lines HepG2, Huh-7, Hep 3b, and LM3 were provided by the Stem Cell Bank of the Chinese Academy of Sciences. All cell lines were cultured in DMEM (Gibco) supplemented with 10% foetal bovine serum (Gibco) and 1% penicillin/streptomycin (Thermo Fisher) at 37 °C with 5% CO2. For shRNA transfection, HCC cells were transfected with the designated IL-6 shRNA plasmids using Lipofectamine 2000 (Invitrogen) to knockdown the expression of IL-6.

### Reagents

NVP-BEZ235 (S1009, Selleckchem) was prepared in DMSO (Gibco) at a concentration of 1 mM. In the experiments, it was further diluted to different working concentrations. For IL-6 neutralization, HCC cells were cultured in the presence of 0.3 μg/mL human IL-6 antibody (MAB206-100, R&D). To block IL-6 activity in vivo, anti-mouse IL-6 (BE0046, BioXcell) was used at a concentration of 200 μg/mouse. The working concentration of NVP-BEZ235 was 100 nM in vitro or 60 mg/kg/day in vivo assay. An IgG2 mouse isotype control antibody (B E0086, BioXcell) was used. Antibodies against total PI3K (4257), phospho-PI3K (4228), total Akt (4691), phospho-Akt (4060), total mTOR (2983), phospho-mTOR (2971), total p70S6K (2708), phospho-p70S6K (9234), cyclin E1 (20808), and cyclin D1 (2978) were purchased from Cell Signalling Technology. S6K1 (ab32359) and phospho-S6K1 (ab131436) were obtained from Abcam.

### Experimental animals

Six- to eight-week-old male C57BL6/J mice were purchased from Beijing Vital River Laboratory Animal Technology Co., Ltd. (Beijing, China). All animals used for the experiments were provided sterilized food and water under constant humidity and temperature. The animal protocols were approved by the Institutional Animal Care and Use Committee of Zhejiang University.

### Murine tumour models

Hydrodynamic tail vein injection (HTVi) and sleeping beauty transposase were combined to create a murine tumour model in the liver. The plasmids (PT3-EF1a-C-Myc, PT/Caggs-NRas-V12, pCMVSB11) were gifts from Dr. Liang Wen at Zhejiang University. The standard procedure comprises a lateral tail vein injection of sterile-filtered PBS, which is equal to 10% of the animal’s weight, and all plasmid DNA is diluted in PBS.

### Cell proliferation assays

Cell proliferation was assessed using the Cell Counting Kit-8 (CCK-8) assay and EdU assay. The CCK-8 and EdU assay kits were all purchased from Beyotime Biotechnology Company. HCC cells treated with drugs were cultured in 96-well plates for the indicated times. After adding 10 μL of CCK-8 solution to each well, the absorbance value (OD) at 450 nm was measured using a microplate reader. The percentage of cell viability was calculated according to the absorbance.

EdU cell proliferation was assessed according to the manufacturer’s instructions. Briefly, HCC cells were seeded into six-well plates and treated with drugs. Then, 10 μM EdU was added and cultured for 2 h. The cells were fixed in 4% paraformaldehyde and permeabilized using 0.3% Triton X-100. Finally, the cells were washed in PBS and stained with Hoechst 33342. The samples were imaged using a fluorescence microscope and analyzed using ImageJ software.

### Cell cycle analysis

After harvesting the samples, the cells were fixed in 70% cold ethanol for 2 h at 4 °C and stained with propidium iodide staining solution for 30 min at 37 °C protected from the light. The results were analyzed using a flow cytometer and FlowJoV10.7.1 software for further analysis.

### Western blot analysis

Cells or liver tissues were lysed in RIPA buffer (Thermo Fisher Scientific) with a protease inhibitor (B14001; Bimake) and a phosphatase inhibitor cocktail (B15001; Bimake). Equal concentrations of proteins were separated on 8–12% SDS-PAGE gels and then blotted onto PVDF membranes (Millipore, USA). After blocking with 5% bovine serum albumin, the blots were incubated with primary antibodies, including AKT (ab18785, Abcam), phospho-AKT (ab38449, Abcam), mTOR (2972, CST), phospho-mTOR (2971; CST), total p70S6K (9202, CST), phospho-p70S6K (9205, CST)) and β-actin (PTM-1012, PTM BIO), overnight at 4 °C. All membranes were then incubated with horseradish peroxidase-conjugated secondary antibodies. Finally, immunoreactions were detected using ECL reagents (Thermo Fisher Scientific, USA), and band intensity was quantified using ImageJ.

### Cell invasion and migration assay

Transwell assays were performed to assess the migration and invasion ability of samples. HCC cells in FBS-free medium were seeded into the upper chambers, which were precoated with Matrigel, and 10% foetal bovine serum medium was added into the lower chambers. Then, the cells were cultured at 37 °C for 24 h, and the Transwell chamber was removed. After fixation in 4% paraformaldehyde for 30 min and staining with 0.1% crystal violet for 15 min at room temperature, the cells were imaged and quantified under a microscope.

### Haematoxylin and eosin staining (HE) and immunohistochemistry (IHC)

Analysis of histopathological changes in liver cancer was performed according to the standard HE staining procedure. Liver samples were obtained and fixed in 10% formalin (SF98-4; Fisher) overnight at room temperature. Then, the tissues were dehydrated, embedded in paraffin, and cut into 5 mm-thick sections. According to the manufacturers’ procedures, sections were stained with haematoxylin-eosin. Additionally, IHC analysis for IL-6 and Ki-67 was also performed in liver samples. Sections were incubated with goat anti-mouse IL‑6 polyclonal antibody (R&D) at 5 µg/mL for 1 h at room temperature and antibodies specific to Ki-67 (Abcam) at 10 µg/mL overnight at 4 °C. The numbers or areas of IL-6+ and Ki-67+ cells were quantified by morphometry or manual counting.

### Enzyme-linked immunosorbent assay

The HCC cell lines were pre-treated with NVP-BEZ235 (100 nM) for 24 h. The culture supernatant was separated from the cell by centrifugation at 2000× rpm for 20 min. The expression levels of IL-1β, IL-6, IL-18, TNF-α, IFN-γ, and TGF-β were determined by commercial ELISA kits (Enzyme-linked Biotechnology Co., Ltd., Shanghai, China).

### Immunofluorescence

Hep 3b and Huh-7 cell lines were washed three times with phosphate-buffered saline (PBS), and then fixed with 4% paraformaldehyde for 20 min, washed three times with PBS. Then the two cell lines were permeabilized with permeabilizing buffer for 15 min. Goat serum was used for blocking for 30 min. Subsequently, the primary antibodies: anti-dsRNA monoclonal antibody J2 (1:250; SCICONS, Hungary) was applied. After incubating with fluorescent secondary antibodies, the slides were visualized using a confocal microscope (Zeiss LSM 800).

### Statistical analysis

Data were analyzed using GraphPad Prism 8.0.2 software (La Jolla, USA) for experimental data analysis. Differences in mean values between two groups were analyzed using two-tailed Student’s t-tests. *P* < 0.05 was considered statistically significant.

## Results

### The PI3K/AKT/mTOR pathway is active in hepatocellular carcinoma

**Fig. 1:**
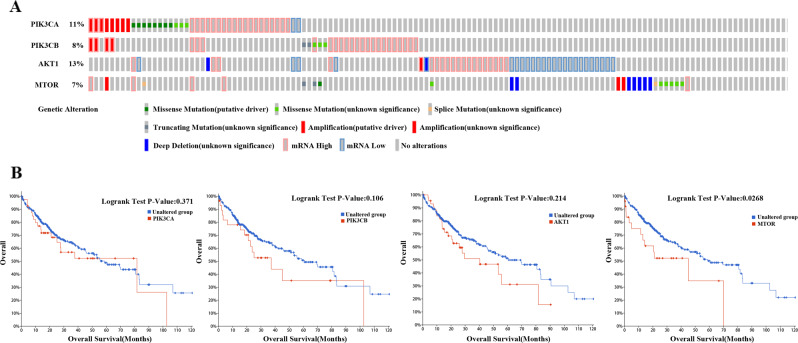
The PI3K/AKT/mTOR pathway is active in hepatocellular carcinoma. **A** Oncoprinter showed the distribution and frequency of PI3K/AKT/mTOR pathway-related gene mutations, copy number changes, and gene expression levels in liver cancer samples. **B** Analysis of survival differences in patients with liver cancer with PI3K/AKT/mTOR gene mutations from TCGA database (data source: https://www.cbioportal.org/).

Aberrant mutations in the PI3K/AKT/mTOR signalling pathway are widely observed in human malignant tumours [23]. Current studies have shown that aberrant mutations in the PI3K/AKT/mTOR signalling pathway are closely related to the development and prognosis of cancer. Abnormal activation of the PI3K/AKT/mTOR pathway is a typical pathway in the pathogenesis of HCC [24]. In this study, querying the TCGA database, we analyzed the genetic characteristics of the PI3K/AKT/mTOR signalling pathway in HCC patient samples. The oncoprinter plot indicated that the expression of PIK3CA, PIK3CB, AKT1, and mTOR genes in HCC patients harboured point mutations, copy number changes, and abnormal gene expression in key genes of the PI3K/AKT/mTOR pathway (Fig. 1A). Furthermore, a survival analysis based on aberrant PI3K/AKT/mTOR signalling revealed poor survival of HCC patients (Fig. 1B). In order to explore the effect of these gene mutations on the survival differences in HCC patients, the human Protein Atlas (https://www.proteinatlas.org/) was used to reconfirm our experiments. The expression and survival differences of PIK3CA, PIK3CB, AKT1, mTOR, and IL-6 in liver cancer patients were analyzed in the Supplementary Figs. 1 and 2.

### NVP-BEZ235 inhibits HCC cell proliferation in a dose- and time-dependent manner

**Fig. 2:**
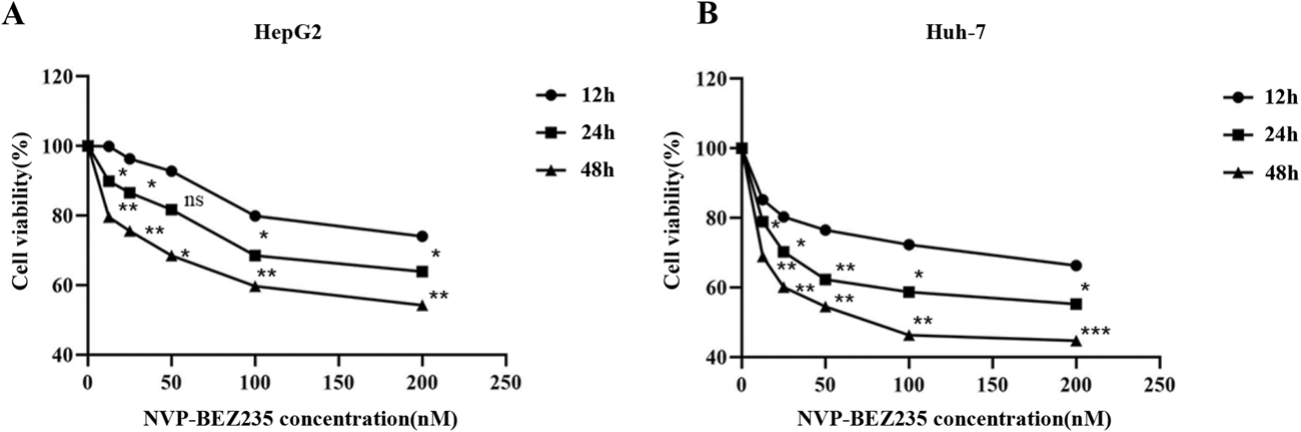
NVP-BEZ235 inhibits HCC cell proliferation in a dose- and time-dependent manner. **A** The proliferation rate of HepG2 cells treated with NVP-BEZ235. **B** The proliferation rate of Huh-7 cells treated with NVP-BEZ235. Compared to the 12 h treatment group. ns ≥ 0.05, * *P* < 0.05, ** *P* < 0.01, * *P* < 0.001.

We used CCK-8 reagent to determine the effect of NVP-BEZ235 on the proliferation of HCC cells. The concentration gradient of NVP-BEZ235 was 0, 12.5, 25, 50, 100, and 200 nm. HepG2 and Huh-7 cells were treated for 12, 24, and 48 h, and then the cell survival rate was calculated to evaluate cell proliferation activity. In order to explore the appropriate concentrations of NVP-BEZ235, the IC50 of NVP-BEZ235 in four cell lines include HepG2 cell line, Huh-7 cell line, Hep 3b cell line, and LM3 cell line were carried out independently (Supplementary Fig. 3). The appropriate concentration of NVP-BEZ235 (100 nM) in vitro was applied for subsequent experiments. The results showed that the survival rate of HCC cells decreased with increasing NVP-BEZ235 concentration and with the extension of time, as shown in Fig. 2A, B. These results indicate that NVP-BEZ235 inhibits the proliferation of HCC cells in a dose- and time-dependent manner.

### NVP-BEZ235 increases the expression of IL-6

**Fig. 3:**
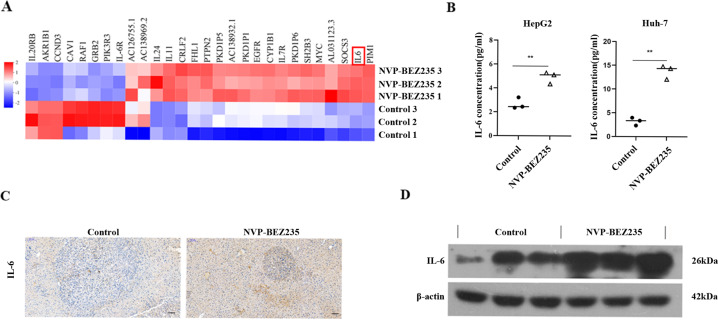
NVP-BEZ235 increases the expression of IL-6. **A** RNA-seq-derived transcript analysis of NVP-BEZ235-induced genes in HepG2 cells. There was at least a two-fold up- or downregulation in the gene (*P* < 0.01). Red indicates higher expression, and blue indicates lower expression. **B** IL-6 protein levels in HCC cells measured by ELISA. **p < 0.01. **C** Representative images of IL-6 immunohistochemical staining in the control group and NVP-BEZ235-treated group from mouse liver tumour tissues. 100×; scale bar, 100 μm. **D** IL‐6 protein expression in the control group and NVP-BEZ235-treated group by western blotting of mouse liver tumour tissues.

Many patients who are administered PI3K inhibitors experience adverse reactions, such as pneumonia, colitis, and immunotoxicity, as described in previous clinical reports. Drug toxicity is a common challenge for small molecule inhibitors. To further explore the potential effect of NVP-BEZ235, we performed transcriptome analysis using RNA-Seq. The heat map shows several key differentially expressed genes (Fig. 3A). Here, we found that the relative expression of the IL-6 gene was significantly upregulated in HepG2 cells compared to the control group. ELISA results revealed that compared to the untreated group, expression of the IL-6 protein increased in HepG2 and Huh-7 cells treated with NVP-BEZ235 (Fig. 3B). Furthermore, we established an HCC model by hydrodynamically transfecting activated forms of C-myc and N-ras oncogenes into the mouse liver. After treatment with NVP-BEZ235, we examined the protein expression of IL-6. Immunohistochemical results demonstrated that the expression of IL-6 in mouse liver tumours in the group treated with NVP-BEZ235 was remarkably increased compared to that in the control group (Fig. 3C). This result was also confirmed by western blot (Fig. 3D).

### IL-6 blockade increases NVP-BEZ235-induced inhibition of cell viability in HCC cells

**Fig. 4:**
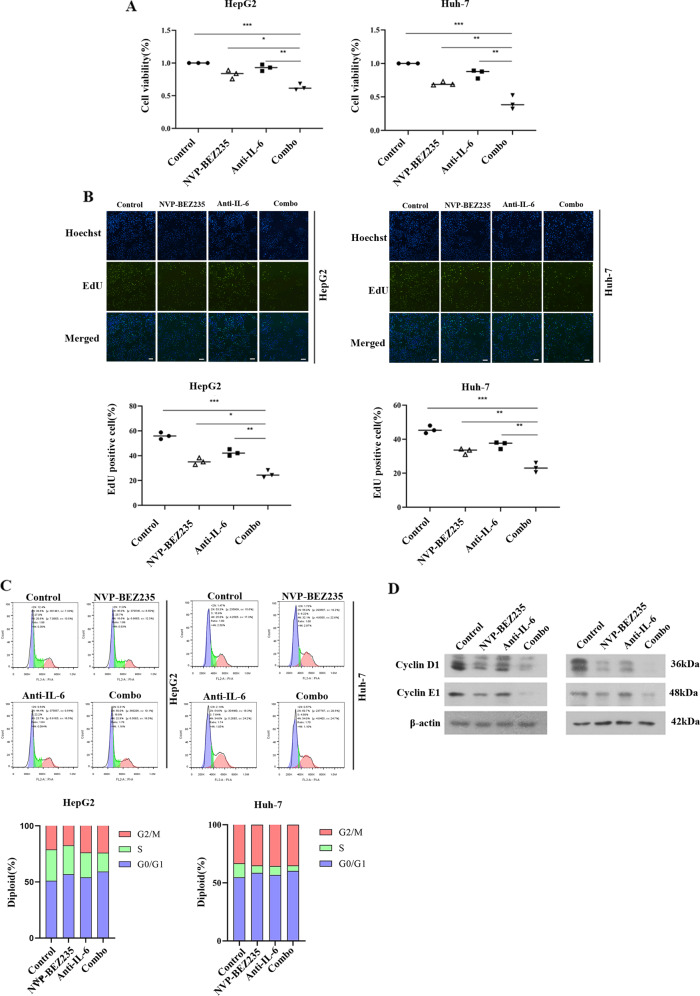
IL-6 blockade increases NVP-BEZ235-induced inhibition of cell viability in HCC cells. **A** Cell proliferation rates of HepG2 and Huh-7 cells treated with NVP-BBEZ235, anti-IL-6 Ab, or their combination for 24 h via CCK-8 assay. **B** The effects of NVP-BBEZ235, anti-IL-6 Ab, or their combination on the proliferation of HepG2 and Huh-7 cells for 24 h via EdU staining assay. **C** Cell cycle profiles of HepG2 and Huh-7 cells treated with NVP-BBEZ235, anti-IL-6 Ab, or their combination for 24 h via flow cytometry analysis. **D** Cyclin D1 and cyclin E1 protein expression was detected by western blotting.

We next explored whether anti-IL-6 combined with NVP-BEZ235 enhanced the inhibition of tumour cell proliferation. After starvation for 6 h, HepG2 and Huh-7 cells were treated with 100 nm NVP-BEZ235 or a neutralizing concentration of 0.3 μg/mL IL-6 neutralizing antibody. The results of the CCK-8 assay showed that compared to the control group, the combination group had significantly reduced cell viability (Fig. 4A). The EdU assay also confirmed that NVP-BEZ235 inhibited the proliferation of HCC cells, and the inhibitory effect was significantly enhanced after inhibiting IL-6 (Fig. 4B). Furthermore, we evaluated whether the combination of NVP-BEZ235 and IL-6 antibody suppressed cell growth by influencing cell cycle progression. Flow cytometry analysis revealed that the percentage of cells in the G0/G1 phase was increased in the combined treatment group (*p* < 0.05), and S phase was reduced (*p* < 0.05), whereas cells in the G2/M phase exhibited no significant changes (på 0.05). Western blot results showed that the combined application of NVP-BEZ235 and IL-6 antibody significantly downregulated protein expression of cyclin D1 and cyclin E1, which are important cell cycle regulatory proteins in G1 phase (Fig. 4C, D). The effect of anti-IL-6 combined with NVP-BEZ235 on the Hep 3b cell lines and LM3 cell lines was shown in the Supplementary Fig. 4. The side effect of increased inflammation after NVP-BEZ235, and the role of IL-6 antibody in reducing inflammation were also evaluated in the Supplementary Fig. 5.

### IL-6 blockade increases NVP-BEZ235-induced inhibition of cell migration and invasion in HCC cells

**Fig. 5:**
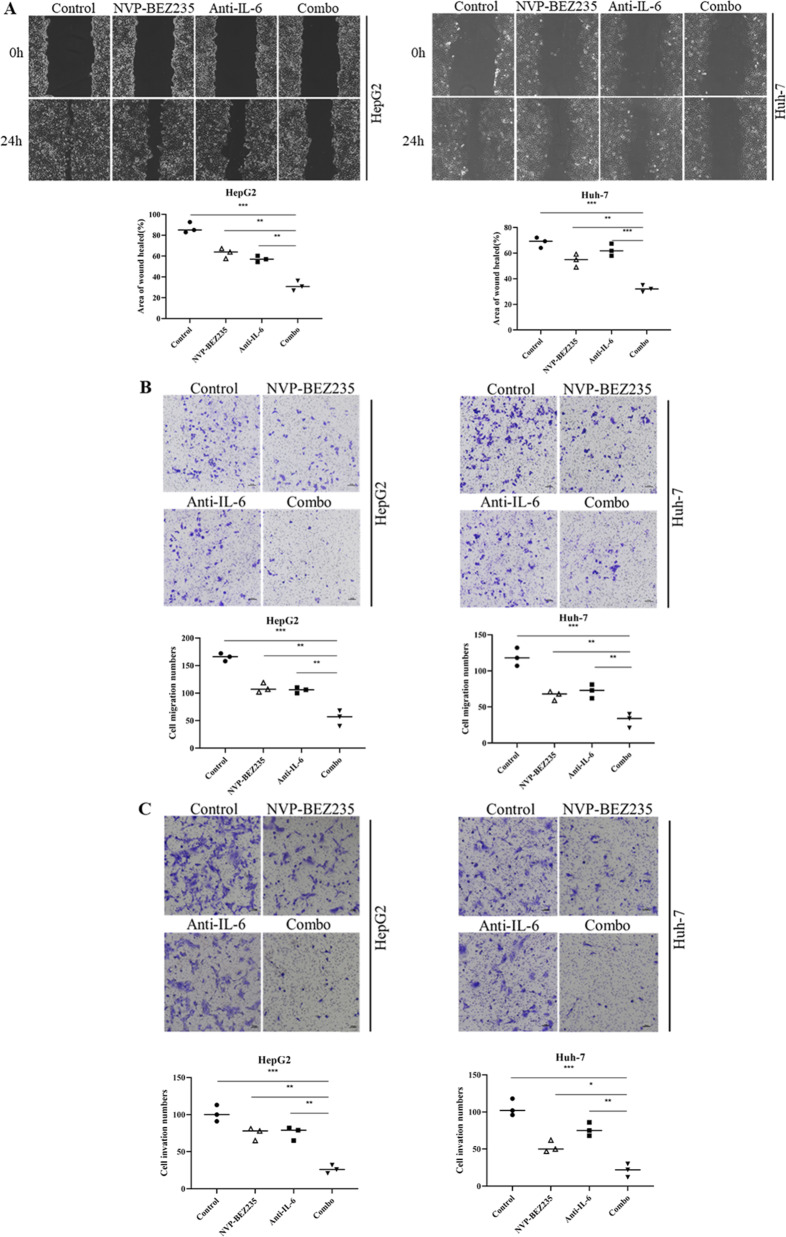
IL-6 blockade increases NVP-BEZ235-induced inhibition of cell migration and invasion in HCC cells. **A** The scratch experiment in response to treatment of HepG2 and Huh-7 cells with NVP-BBEZ235, anti-IL-6 Ab, or their combination for 24 h. **B** Analysis of Transwell migration assay upon treatment of HepG2 and Huh-7 cells with NVP-BBEZ235, anti-IL-6 Ab, or their combination for 24 h. **C** Analysis of Transwell invasion assay upon treatment of HepG2 and Huh-7 cells with NVP-BBEZ235, anti-IL-6 Ab, or their combination for 24 h.

Cell migration was assessed by scratch wound assays and Transwell assays. Compared to the control group treated with DMSO, NVP-BEZ235 and IL-6 antibody treatment alone reduced the migration ability of HCC cells, while the inhibitory effect of NVP-BEZ235 and IL-6 antibody cotreatment on the migration ability of HCC cells was more obvious, indicating that IL-6 blockade enhances the inhibitory effect of NVP-BEZ235 on cell migration (Fig. 5A). The combination of NVP-BEZ235 and IL-6 antibody significantly inhibited the migration of HCC cells and exhibited identical results in the scratch wound assays (Fig. 5B). Transwell invasion experiments were used to evaluate the effect of NVP-BEZ235 combined with IL-6 antibody on cell invasion. Compared to the control group, NVP-BEZ235 and IL-6 antibodies alone inhibited the invasion of HCC cells. The ability of NVP-BEZ235 combined with IL-6 antibody to inhibit the invasion of HCC cells was more significant than that of the single drug group, indicating that cell motility of the single drug group was stronger than that of the combined drug group (Fig. 5C). Collectively, these results illustrate that the combination of NVP-BEZ235 and IL-6 antibody significantly promotes an antitumor effect.

### Inhibition of IL-6 combined with NVP-BEZ235 synergistically suppresses the PI3K/AKT/mTOR pathway in HCC cells

**Fig. 6:**
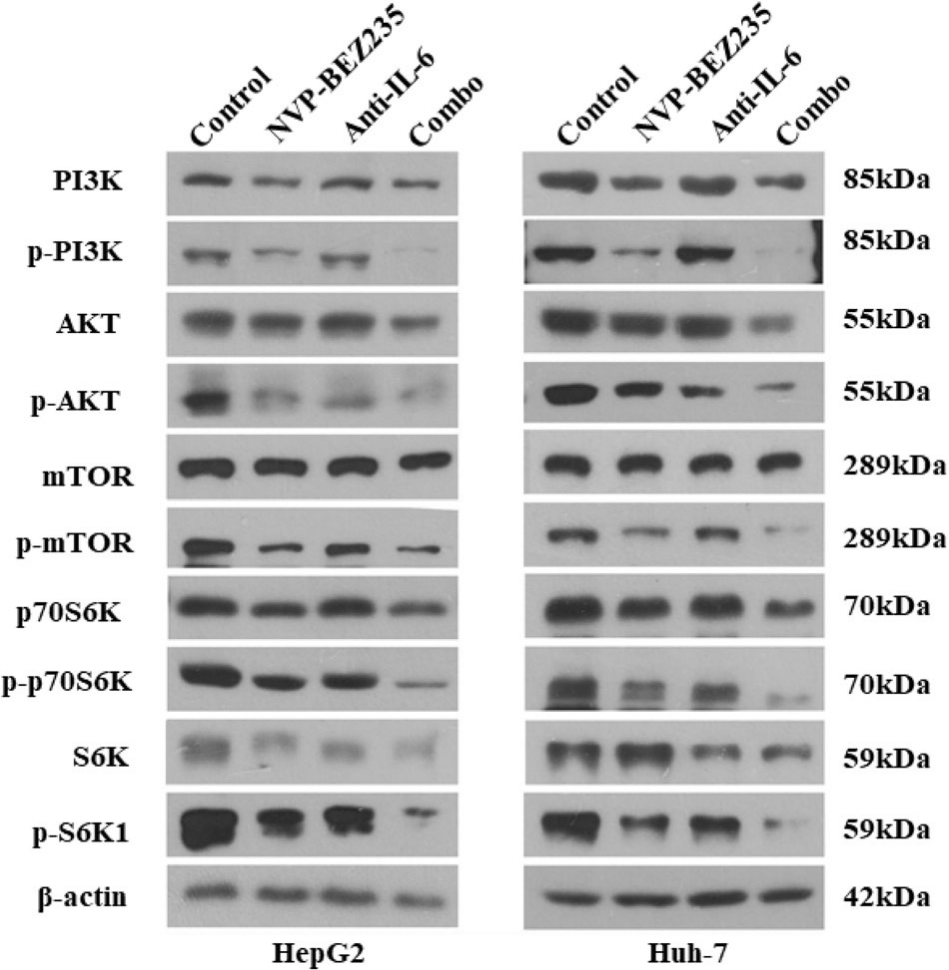
Inhibition of IL-6 combined with NVP-BEZ235 synergistically suppresses the PI3K/AKT/mTOR pathway in HCC cells. Combined treatment inhibited activation of the PI3K/AKT/mTOR pathway in HepG2 and Huh-7 cells. The expression pattern of PI3K/AKT/mTOR pathway-associated proteins was analyzed using western blotting.

Next, key proteins in the PI3K/AKT/mTOR signalling pathway were assessed by western blot assay to further explore the molecular regulatory mechanism of the treatment combination. First, the effect of NVP-BEZ235 on the expression levels of PI3K/mTOR pathway modulators in HepG2 and Huh-7 cell lines was investigated. The PI3K inhibitor LY294002 and the mTORC1 inhibitor rapamycin were used as controls (Supplementary Fig. 6). Then, NVP-BEZ235 was used in HCC cells transfected with the IL-6 shRNA expression vector. It was found that it inhibited the catalytic activity of PI3K, which led to a significant decrease in levels of phosphorylated PI3K (Tyr458/Tyr199). In addition, we found that in the two HCCs, proteins downstream of the PI3K/AKT/mTOR signalling pathway were inhibited in response to treatment with NVP-BEZ235 alone, which resulted in changes in the protein levels of phosphorylated AKT (Ser473) and phosphorylated mTOR (Ser2448), consistent with previous studies. The inhibition of IL-6 expression also significantly reduced the catalytic activity of AKT and mTOR. Expression of phosphorylated P70S6K (thr389) was decreased, which further affected the catalytic activity of the downstream effector S6K protein, leading a significant decrease in the expression of S235/S236P-RPS6 (p-S6K1). Obviously, NVP-BEZ235 acts on the PI3K/AKT/mTOR signalling pathway, and inhibition of IL-6 amplifies this inhibitory effect, enhancing the inhibition. The synergistic effect of targeting the PI3K/AKT/mTOR pathway inhibits the proliferation of HCC cells (Fig. 6).

### NVP-BEZ235 combined with IL-6 antibody effectively inhibits tumour progression and prolongs the survival time of HCC

**Fig. 7:**
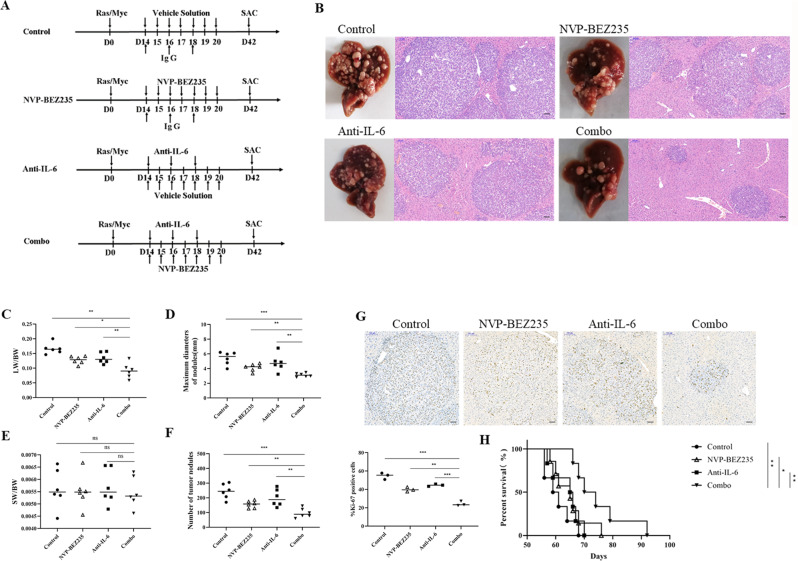
NVP-BEZ235 combined with IL-6 antibody effectively inhibits tumour progression and prolongs the survival time in mice with HCC. **A** Scheme of the experimental procedure for NVP-BBEZ235, anti-IL-6 Ab, or the combination treatment. N-Ras/c-Myc/SB plasmids were transfected into all four groups of mice on Day 0. NVP-BBEZ235 (60 mg/kg) (or vehicle solution) was i.p. injected on Days 14-20, and anti-IL-6 Ab (or isotype IgG) was i.p. injected on Days 14, 16, and 18. All mice were euthanized 6 weeks after oncogene transfection. **B** Gross appearances of livers and H&E-stained liver sections in mice treated with NVP-BBEZ235, anti-IL-6 Ab, or their combination. Magnification, 100×; scale bar, 100 μm. **C**–**F** Tumour loads were evaluated by LW/BW ratios, SW/BW ratios, maximum diameter of nodules, and number of tumour nodules (mm). **G** Left: Representative immunostaining of Ki-67 tumour areas in liver sections. Magnification, 100×; scale bar, 100 μm. Right: Quantification of Ki67+ tumour cell numbers per field. **H** Kaplan–Meier survival curves of overall survival of the four groups of mice. Log-rank test was performed, *n* = 6 per group.

Here, we built a murine HCC model using hydrodynamic transfection in vivo. Mice were randomly divided into four treatment groups: control, NVP-BEZ235 treatment, IL-6 antibody treatment and NVP-BEZ235 combined with IL-6 antibody treatment. Construction of the mouse HCC model and the process of drug treatment are shown in Fig. 7A. Mice were euthanized 6 weeks after transfection for phenotypic analysis. As measured by liver appearance, we found that injection of either NVP-BEZ235 or anti-IL-6 Ab inhibited the tumour progression of HCC to a certain extent but was very limited. However, the combination of NVP-BEZ235 and anti-IL-6 Ab significantly suppressed tumour progression. H&E staining results revealed that there were increased tumour nodules in both the control and single drug groups; however, tumour cell density was markedly reduced in response to the combination of NVP-BEZ235 and anti-IL-6 Ab (Fig. 7B). We also measured the LW/BW ratios, SW/BW ratios, maximal tumour diameters and numbers of tumour nodules as indicator parameters of tumour progression (Fig. 7C–F). Ki-67 immunohistochemical staining was performed to assess tumour cell proliferation. The statistical results revealed that the ratio of Ki-67-positive cells in the liver tumour lesions was 54.70 ± 3.53, 39.92 ± 2.49, 44.61 ± 1.26, and 24.35 ± 2.60 (%) for the control, NVP-BEZ235, anti-IL-6 Ab, and combination groups, respectively (Fig. 7G). We also assessed whether the combination therapy prolongs the survival of tumour mice (Fig. 7H). The mouse survival analysis showed median survival times of 60.2 ± 1.9, 70.5 ± 2.6, 63.7 ± 1.9, and 75.2 ± 4.2 days for the control, NVP-BEZ235, anti-IL-6 Ab and combination groups, respectively. Compared to the control and the two monotherapy therapy groups, the survival time of mice in the combined treatment group was significantly prolonged. Therefore, we confirmed that the combination therapy significantly prolongs survival in tumour-bearing HCC mice.

## Discussion

Hepatocellular carcinoma was currently one of the most fatal malignancy with high incidence rate and mortality. To date, there has been a limited breakthrough in the treatment of HCC due to its complexity and heterogeneity [25]. At present, combination therapy using small molecule inhibitors is gradually becoming a new treatment for HCC [26]. Previous studies have shown that the PI3K/AKT/mTOR signalling pathway plays a key role in the survival and growth of tumour cells, so it might become an ideal intervention target for cancer treatment [27], resulting in the birth of many small molecule targeted drugs. But previous clinical studies have found that many of these drugs are always accompanied by a series of side effects [28], which restricted the further clinical appliance in HCC treatment.

NVP-BEZ235 is a dual PI3K/mTOR inhibitor in phase II clinical trials in advanced cancer patients to study its pharmacokinetics and pharmacodynamics. However, compared with the mTOR inhibitor everolimus, NVP-BEZ235 has been reported with a higher level of IL-6 [22, 29]. As we all know, a large number of clinical samples have shown that the serum IL-6 levels in patients with HCC are significantly higher than that of healthy people, and high serum IL-6 levels are associated with poor prognosis [30]. Thus, we hypothesized that an enhanced the efficacy of NVP-BEZ235 would be observed on the treatment of HCC after blockade of IL-6 with mAb. In this study, we first evaluated the effect of the dual target inhibitor NVP-BEZ235 on HCC, then explored the synergistic effect of IL-6 antibody combined with NVP-BEZ235 for the treatment of HCC. Finally, we propose a combined treatment scheme that provides a theoretical reference for clinical application.

Combined treatment with chemotherapeutic agents and other small molecule inhibitor markedly improved the effects of drug therapy or relieved side effects, which could overcome drug resistance [31, 32]. Generally, chemotherapy would lead to an elevation in several inflammatory cytokines [33, 34]. Cytokines are considered to be the key mediators connecting inflammation and cancer [35]. As shown in the Fig. 3B and Supplementary Fig. 7, we found that in response to treatment with NVP-BEZ235, expression of IL-6 in HCC cells was significantly upregulated, which may lead to an inflammatory reaction in the body. Systemic inflammation reactions and inflammatory cells infiltration are closely related to tumour progression and survival of cancer patients [35]. As the core proinflammatory cytokine in the body, IL-6 has been proven to promote the proliferation, invasion and metastasis of tumour cells, inhibit apoptosis, and promote vascular growth [36]. It also participates in the immune regulation of the tumour microenvironment and promotes the development of tumours [37]. To further explore the mechanism by which NVP-BEZ235 increased the expression of IL-6, the double-stranded RNA was detected by immunofluorescence. Double-stranded RNA was a pathogen-associated molecular patterns and a landmark product when virus-infected animal cells. The current research also found that endogenous double-stranded RNA may be produced during inflammatory response and chemotherapy [38, 39]. It was commonly accepted that the IL-6 was induced by double-stranded RNA [38, 40]. As shown in the Supplementary Fig. 8, the double-stranded RNA was significantly increased in the NVP-BEZ235 treatment group, compared with the control group, which may explain the larger number of IL-6 that was observed in NVP-BEZ235 treat group. Therefore, IL-6 antibody combined with NVP-BEZ235 may further enhance the inhibitory effect of NVP-BEZ235 on HCC progression.

Potential cumulative effects or synergistic effects can be observed in cancer treatment but can also be accompanied by certain toxicities and side effects. With our experiments, we have shown that IL-6 antibody combined with NVP-BEZ235 can enhance the effectiveness of the NVP-BEZ235, minimize drug resistance and diminish toxicity issues and side effects. First, the application of PI3K/AKT/mTOR pathway inhibitors exhibits dose-limiting toxicity [31]. Combined treatment with monoclonal antibodies can avoid the superimposed toxicity between chemotherapy drugs for some dysfunctional parts of the signal transduction pathway [41, 42]. Second, because the inhibitors themselves may destroy the negative feedback mechanism in the body, interfering with or reduce the therapeutic effect of PI3K/AKT/mTOR pathway inhibitors, their use can result in the emergence of drug resistance in the body. However, the development of effective targeted drug combinations can overcome the occurrence of drug resistance in the course of drug use and increase drug sensitivity [43, 44]. As for the classic JAK/STAT3 signalling pathway, the combination of IL-6 antibody and NVP-BEZ235 also seem lead to stronger inhibitory effects (Supplementary Fig. 9). As report by Vogt PK, the PI3K-mTOR and STAT3 signalling pathways may exist a functional link that warrant further exploration on the potential underlying biological mechanisms [45]. Finally, considering the interaction of pharmacokinetics, molecular targeted drugs combined with monoclonal antibodies may display the advantages of reduced toxicity, fewer side effects, and stronger efficacy [46]. A working model in the Supplementary Fig. 10 was provided to make readers easier to understand the context of the manuscript. This study may provide new ideas for enhancing the drug sensitivity of small molecule inhibitors and reducing their side effects, and the specific regulatory mechanism will be further explored in future work.

## Supplementary information

checklist_2

Supplemental Fig. 1

Supplemental Fig. 2

Supplemental Fig. 3

Supplemental Fig. 4

Supplemental Fig. 5

Supplemental Fig. 6

Supplemental Fig. 7

Supplemental Fig. 8

Supplemental Fig. 9

Supplemental Fig. 10

## Data Availability

All data generated in the study are included in this article.
