# Supplementary figures and images for: The synergistic antitumor effect of IL-6 neutralization with NVP-BEZ235 in hepatocellular carcinoma
Source: Cell Death Dis. 2022 Feb 14;13(2):146. doi: 10.1038/s41419-022-04583-5 (PMC8844296; doi:10.1038/s41419-022-04583-5)

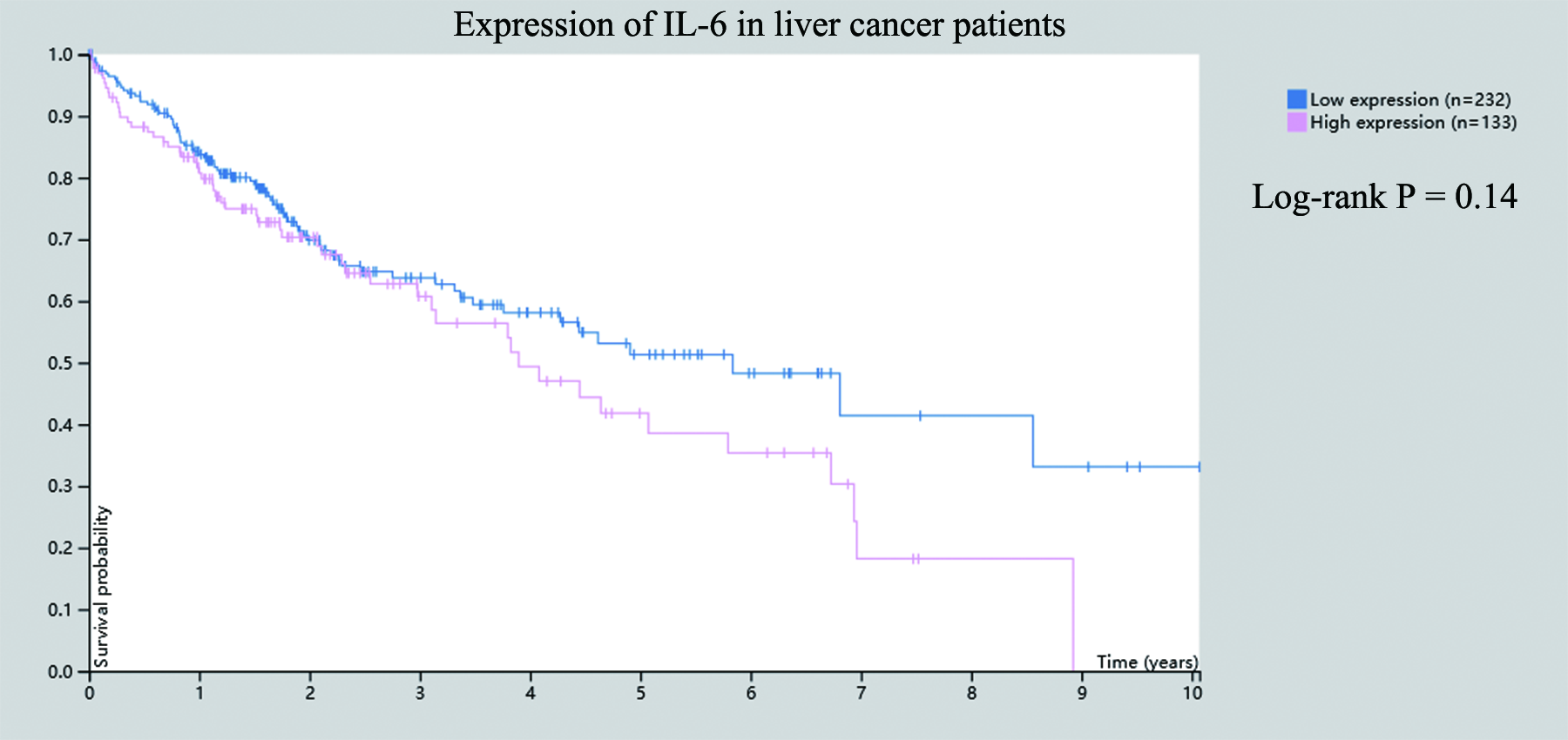

Supplement: Supplementary file 2 — Supplemental Fig. 1 [file 41419_2022_4583_MOESM2_ESM.tif]

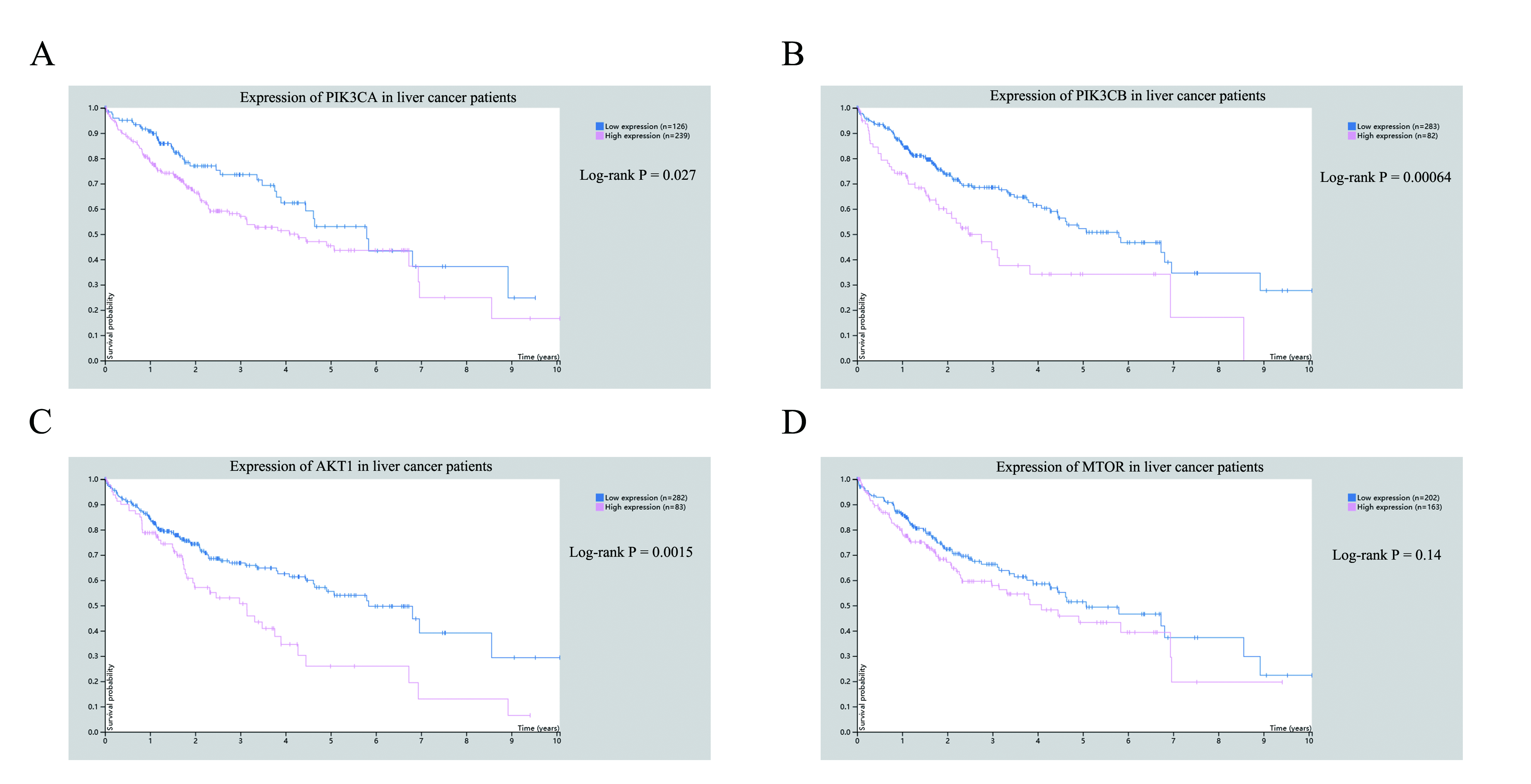

Supplement: Supplementary file 3 — Supplemental Fig. 2 [file 41419_2022_4583_MOESM3_ESM.tif]

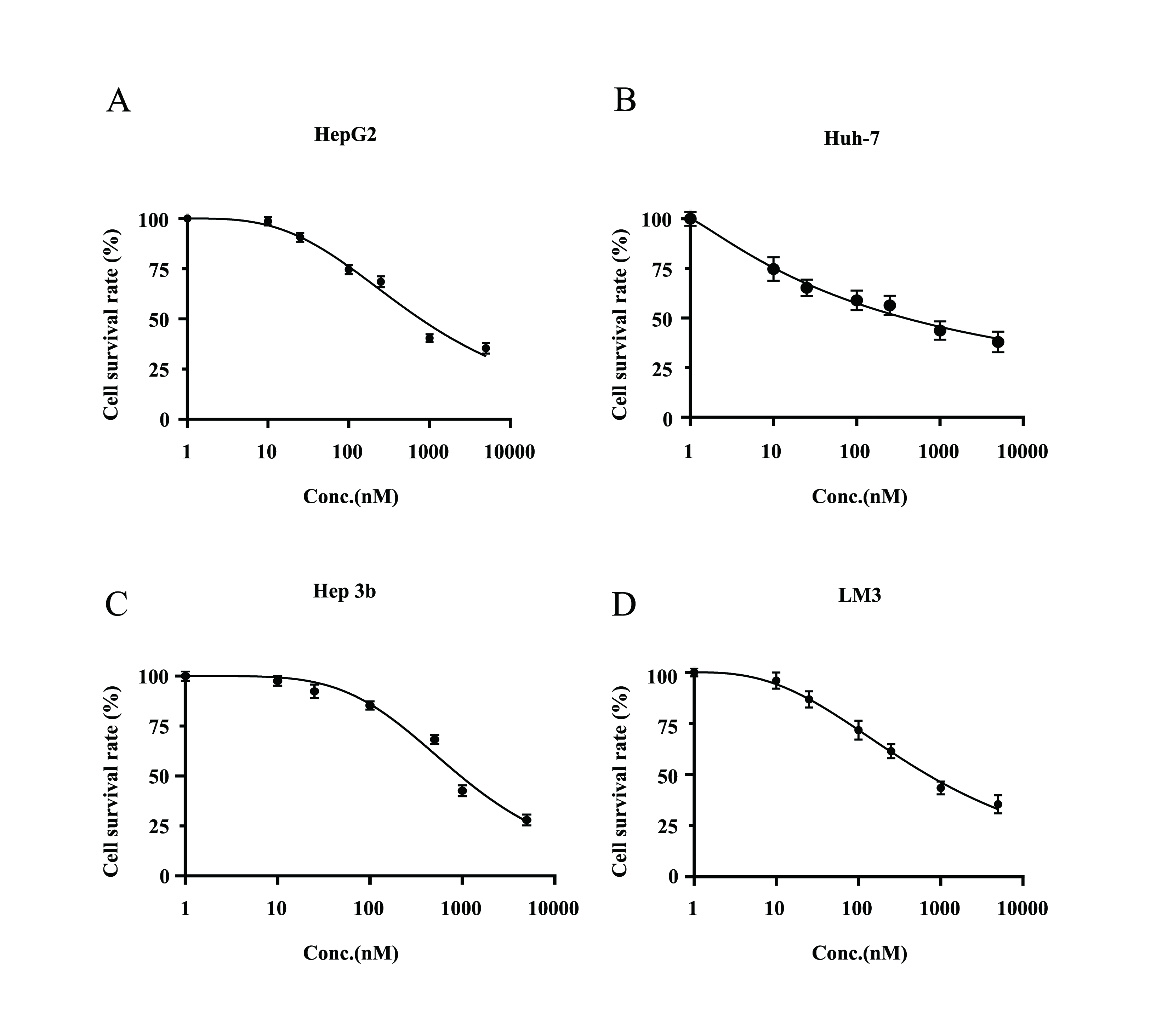

Supplement: Supplementary file 4 — Supplemental Fig. 3 [file 41419_2022_4583_MOESM4_ESM.tif]

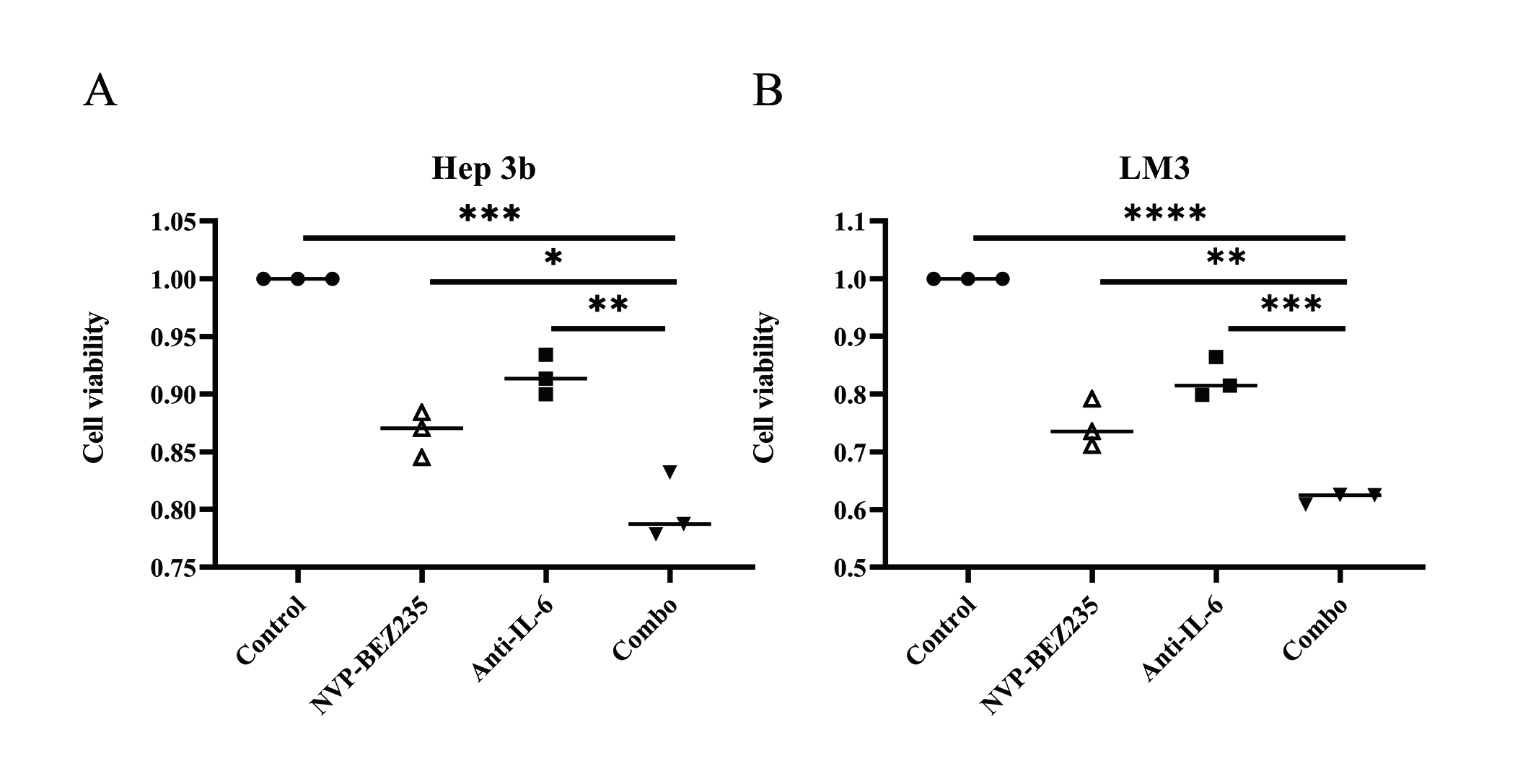

Supplement: Supplementary file 5 — Supplemental Fig. 4 [file 41419_2022_4583_MOESM5_ESM.tif]

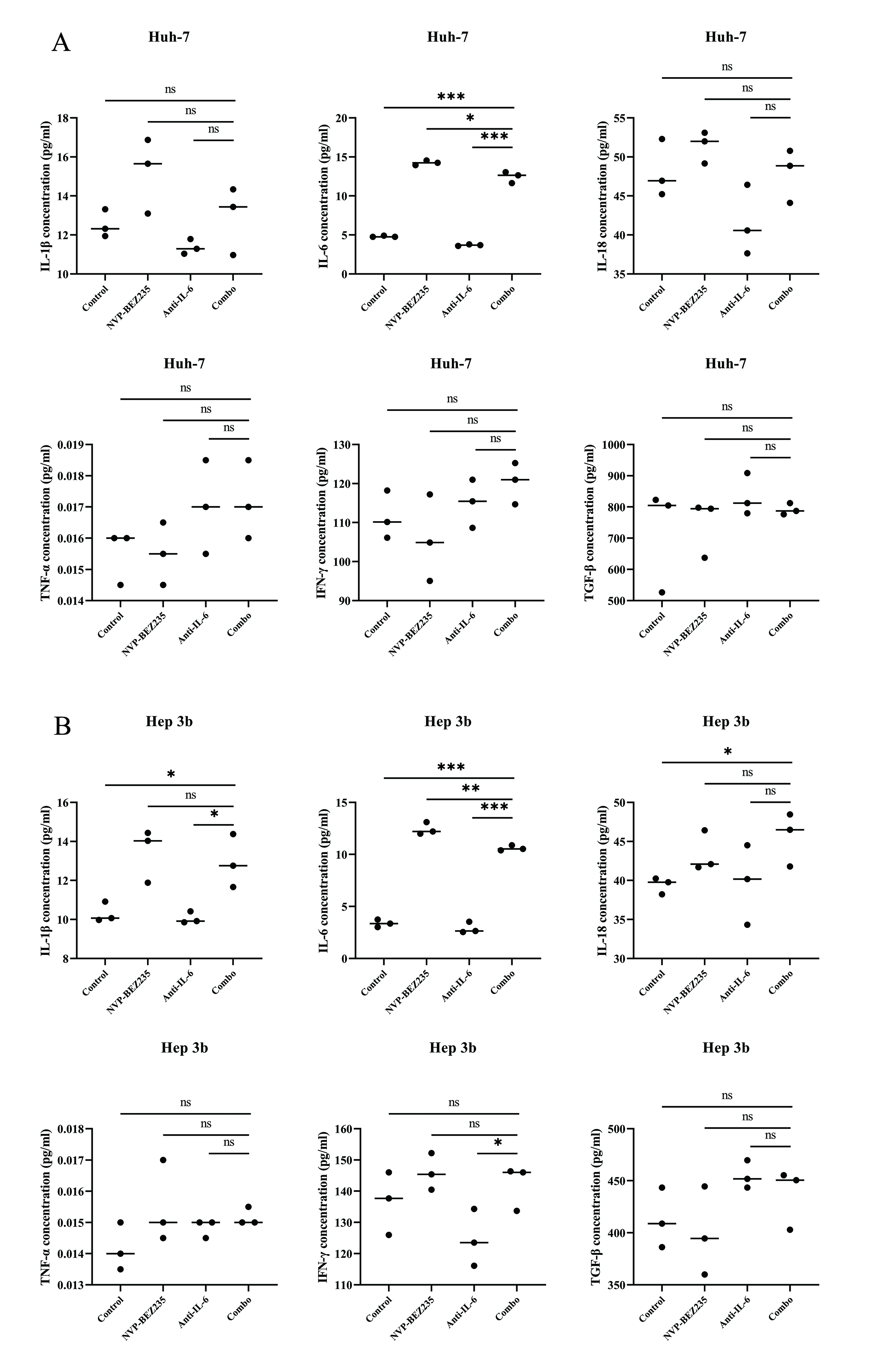

Supplement: Supplementary file 6 — Supplemental Fig. 5 [file 41419_2022_4583_MOESM6_ESM.tif]

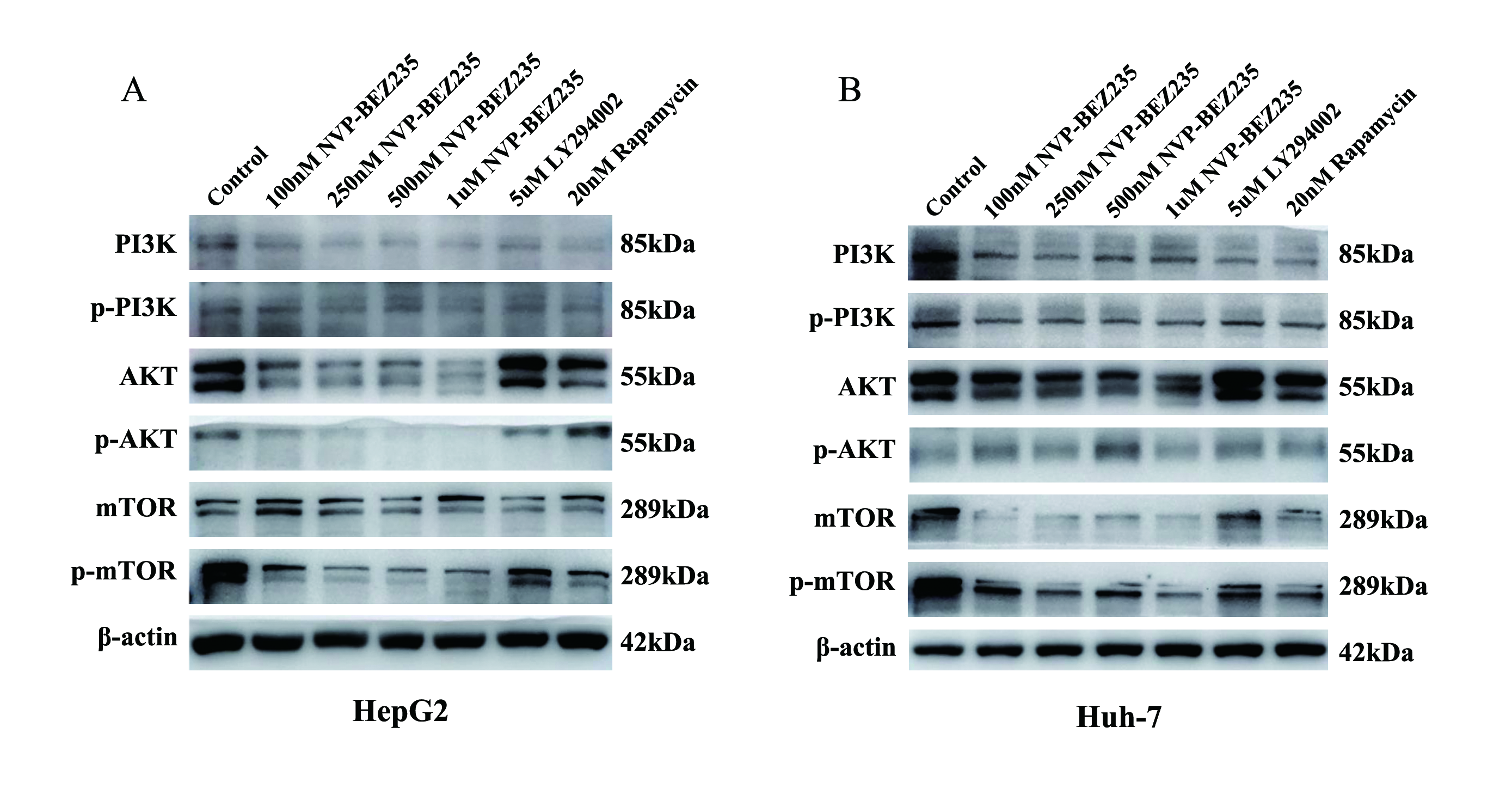

Supplement: Supplementary file 7 — Supplemental Fig. 6 [file 41419_2022_4583_MOESM7_ESM.tif]

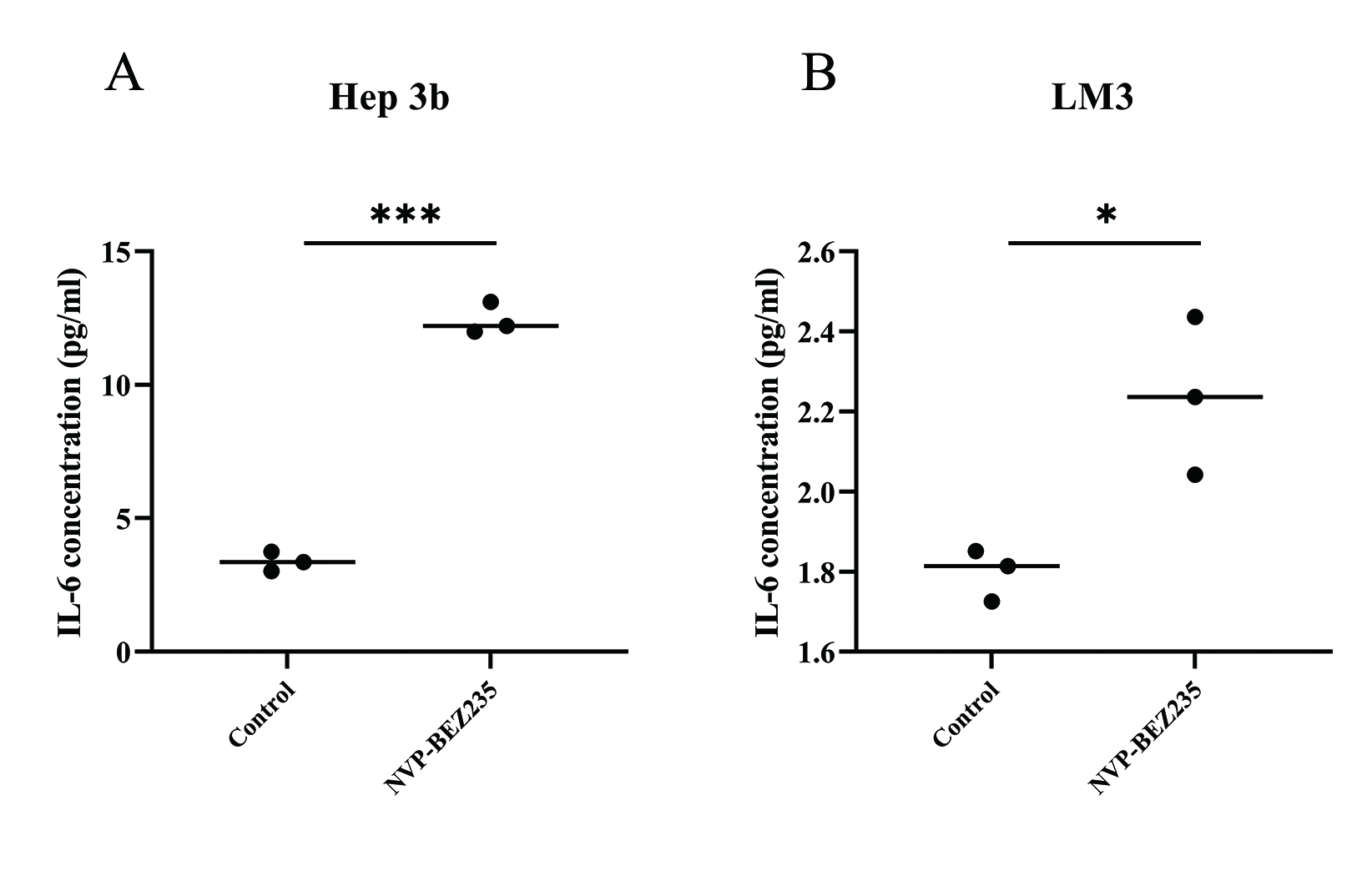

Supplement: Supplementary file 8 — Supplemental Fig. 7 [file 41419_2022_4583_MOESM8_ESM.tif]

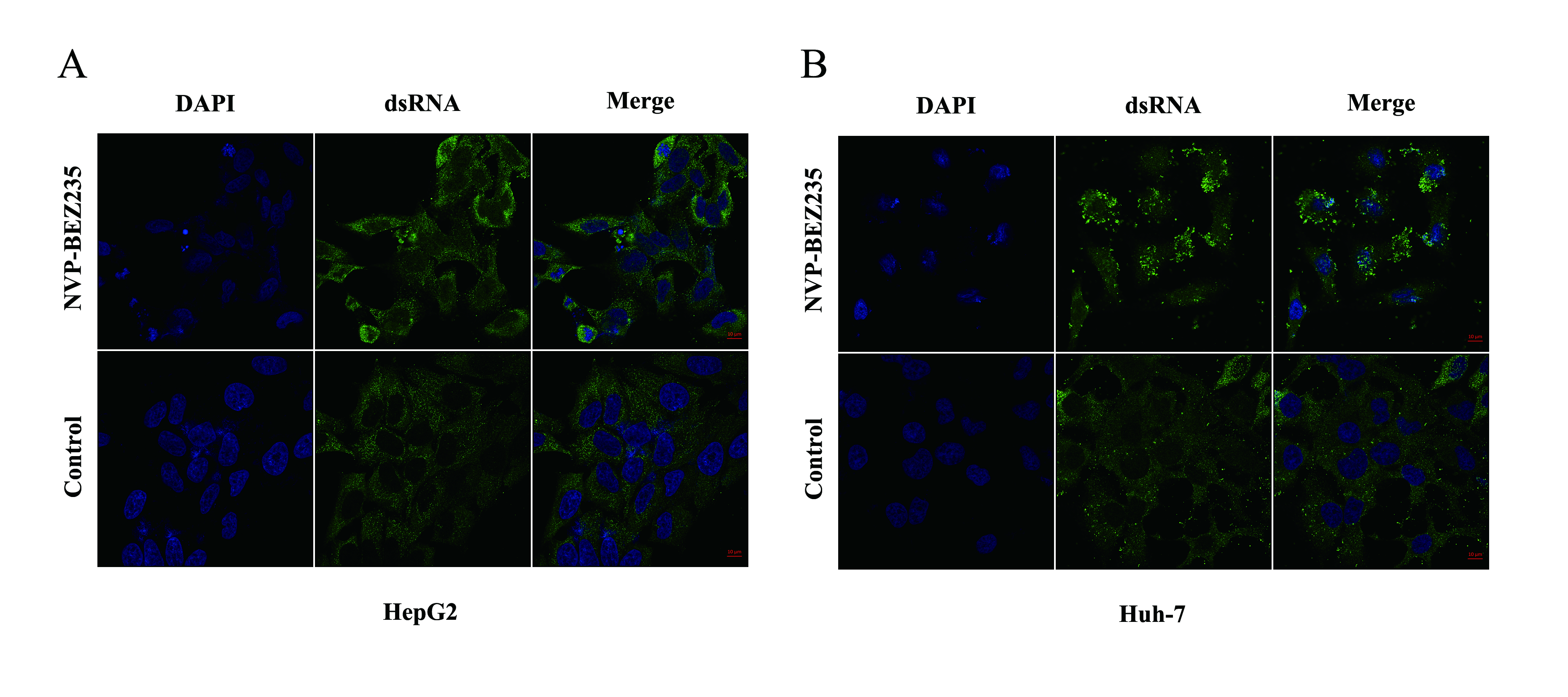

Supplement: Supplementary file 9 — Supplemental Fig. 8 [file 41419_2022_4583_MOESM9_ESM.tif]

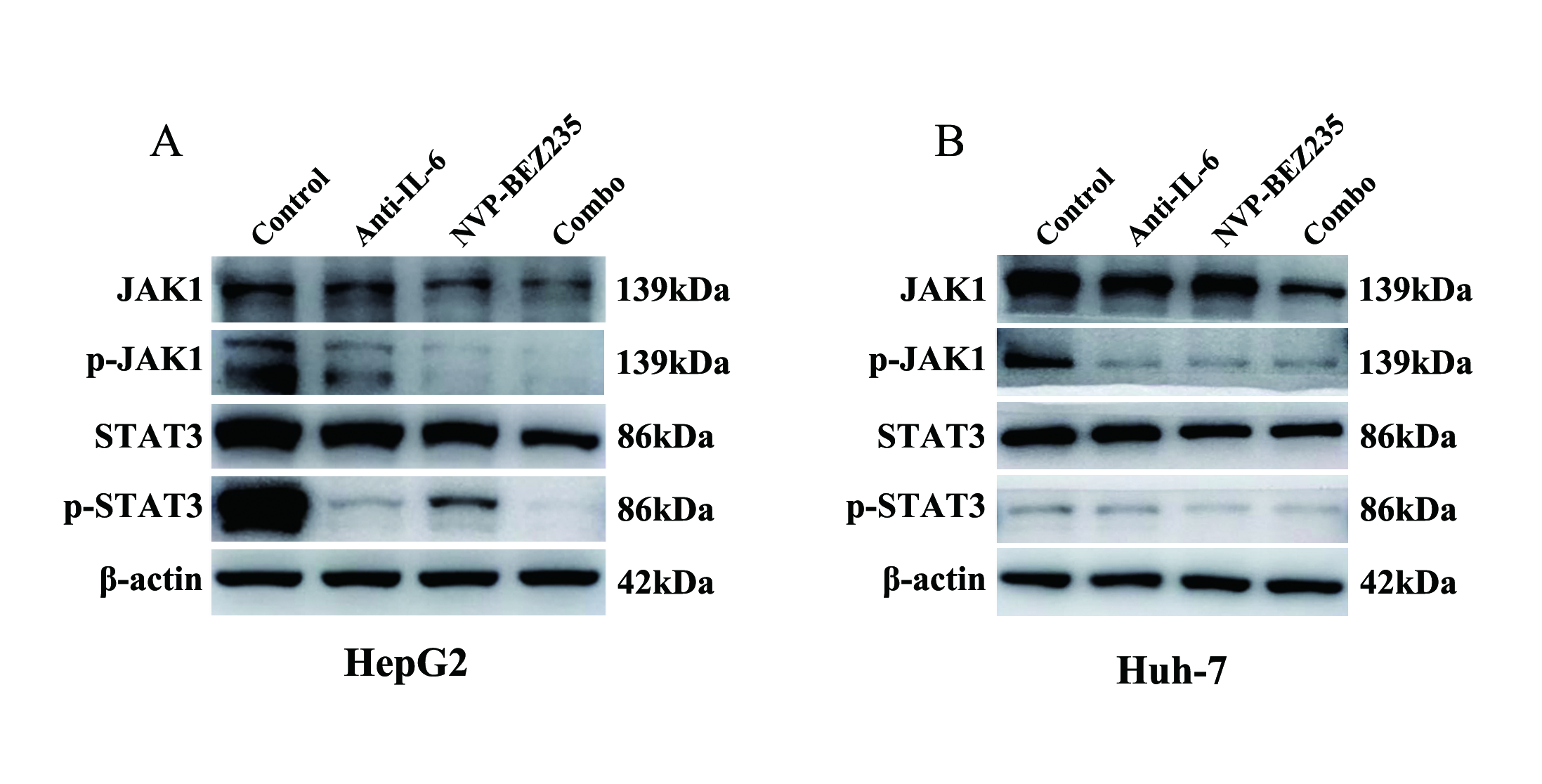

Supplement: Supplementary file 10 — Supplemental Fig. 9 [file 41419_2022_4583_MOESM10_ESM.tif]

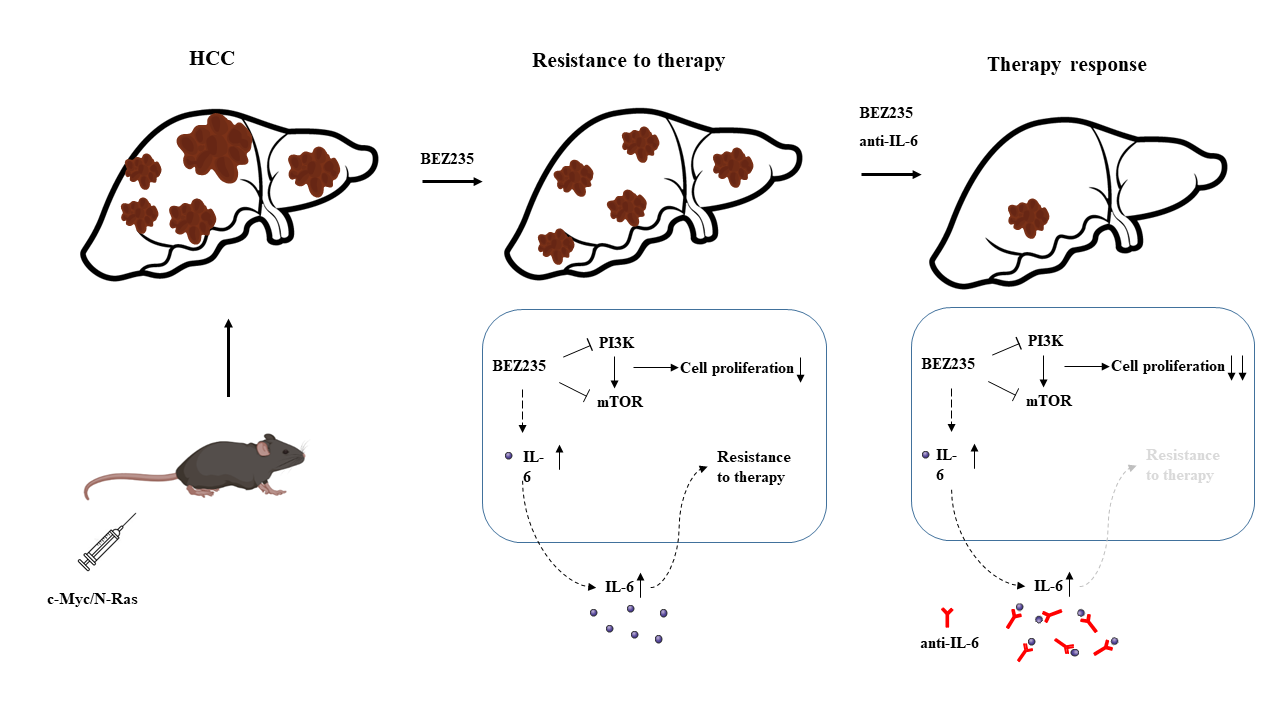

Supplement: Supplementary file 11 — Supplemental Fig. 10 [file 41419_2022_4583_MOESM11_ESM.tif]
